# The first complete chloroplast genome of *Tetradium daniellii* (Benn.) T. G. Hartley

**DOI:** 10.1080/23802359.2021.1990151

**Published:** 2022-01-24

**Authors:** Mei Yu, Ke-xue Yu, Yong-jun Zhao, Jian-quan Tang, Yizeng Lu, Lei Wang

**Affiliations:** a College of Food Science and Engineering, Shandong Agriculture and Engineering University, Ji’nan, P. R. China; b Shandong Provincial Center of Forest and Grass Germplasm Resources, Ji’nan, P. R. China; c Shandong Provincial Engineering Laboratory for Utilization of Woody Oil Resources, Ji’nan, P. R. China; d Hunan University of Humanities and Science, Loudi, Hunan Province, P. R. China

**Keywords:** ：*Tetradium daniellii*, Illumina, complete chloroplast, phylogenetic

## Abstract

*Tetradium daniellii* (Benn.) T. G. Hartley is an important medicinal, ornamental, and timber tree species and belongs to genus *Tetradium* in family of Rutaceae. It is widely distributed in warm temperate deciduous broad-leaved forest areas in northern China, Korean Peninsula and Japan. In this study, we sequenced its sample and determined complete chloroplast genome. The CP genome of *T. daniellii* has a circle structure with the length 158,446 bp, includes a small single copy region (17, 972 bp), a large single copy (86, 478 bp) and two inverted repeats (26,998 bp). There were 131 genes, which included 86 protein-coding genes, 8 rRNA and 37 tRNA, and overall GC content covered by 38.3%. The gene trnK-UUU, *rps*16, trnG-UCC, *atp*F, *rpo*C1, trnL-UAA, trnV-UAC, *pet*B, *pet*D, *rpl*16, *rpl*2, *ndh*B, trnI-GAU, trnA-UGC and *ndh*A contained an intron; gene *clp*P, *ycf*3 contained 2 introns. The phylogenetic result showed that *T. daniellii* had the closest relationship with *Tetradium ruticarpum* (NC_052830).

*Tetradium daniellii* (Benn.) T. G. Hartley is a plant of the genus *Tetradium* in the family *Rutaceae*, which is widely distributed in warm temperate deciduous broad-leaved forest areas in northern China, Korean Peninsula, and Japan, at present, the research of *T. daniellii* mainly focuses on Chemical Components (Yoo et al 2002; Lee et al 2011; Li et al 2013). However, we can hardly find the research about the complete chloroplast of *T. daniellii*, and in NCBI we can not find the record of the complete chloroplast genome sequence of it before this study. So in this study, we sequenced the sample and determined the complete chloroplast genome, then we did the phylogenetic analysis to determine the position in family *Rutaceae*.

The sample of *T. daniellii* was from Taishan Mountain, Taian, Shandong Province, China (N37°11′50″, E121°40′50″), we only collected several stems and leaves of the sample but not the whole plant. We used part of leaves to extract the DNA and stored others in National Plant Specimen Resource Center (http://www.cvh.ac.cn/, barcode SDF1004500, Lei Wang, Email: stoawang@126.com). The fresh leaves were used to extract the total genomic DNA with the modified CTAB method (Doyle and Doyle 1987), then we constructed the library with an average length of 350 bp using the NexteraXT DNA Library Preparation Kit (Illumina, San Diego, CA) and sequenced by Illumina Novaseq 6000 platform. The 1.57 Gb clean sequence data was assembled by *de novo* assembler SPAdes v3.11.0 (Bankevich et al. 2012) with the reference genome *Phellodendron amurense* (NC_035551). Finally the complete chloroplast genome was annotated by PGA (Qu et al. 2019). We submitted the assembled genome DNA to GenBank under the accession number of MZ145060, and SRA submitted to NCBI under the BioProject No. PRJNA732282 and SRA number: SRR14663312

The chloroplast genome of *T. daniellii* has a circle structure with the size of 158,446 bp in length that contains a large single copy (LSC: 86,478 bp) region, a small single copy (SSC: 17,972 bp) and two inverted repeats (IRs: 26,998 bp) region. The overall GC content was 38.3%. There were 131 genes including 86 protein-coding genes, 37 tRNA and 8 rRNA. Each of *trn*K-UUU, *rps*16, *trn*G-UCC, *atp*F, *rpo*C1, *trn*L-UAA, *trn*V-UAC, *pet*B, *pet*D, *rpl*16, *rpl*2, *ndh*B, *trn*I-GAU, *trn*A-UGC and *ndh*A contained one intron respectively, *clp*P and *ycf*3 had two introns respectively. While *rps*12 had Trans splicing.

To determine the phylogenetic position of *T. daniellii* in family *Rutaceae*, we selected 19 complete chloroplast genomes from NCBI and aligned with *T. daniellii* by using Mafft 7.473 (Katoh and Standley 2013) with strategy of FFT-NS-2. Then we used model finder to selecte TVM + F + I + G4 model (Subha et al. 2017) and constructed the phylogenomic tree (Figure 1) by IQtree 2.0 (Minh et al. 2020) with 1000 bootstrap and Maximum-likehood method. During the ML tree construction, the complete chloroplast genomes of *Murraya koenigii* (MT806177) was used as outgroup. Then the result showed that *T. daniellii* had the closest relationship with *Tetradium ruticarpum* (NC_052830)

**Figure 1.**
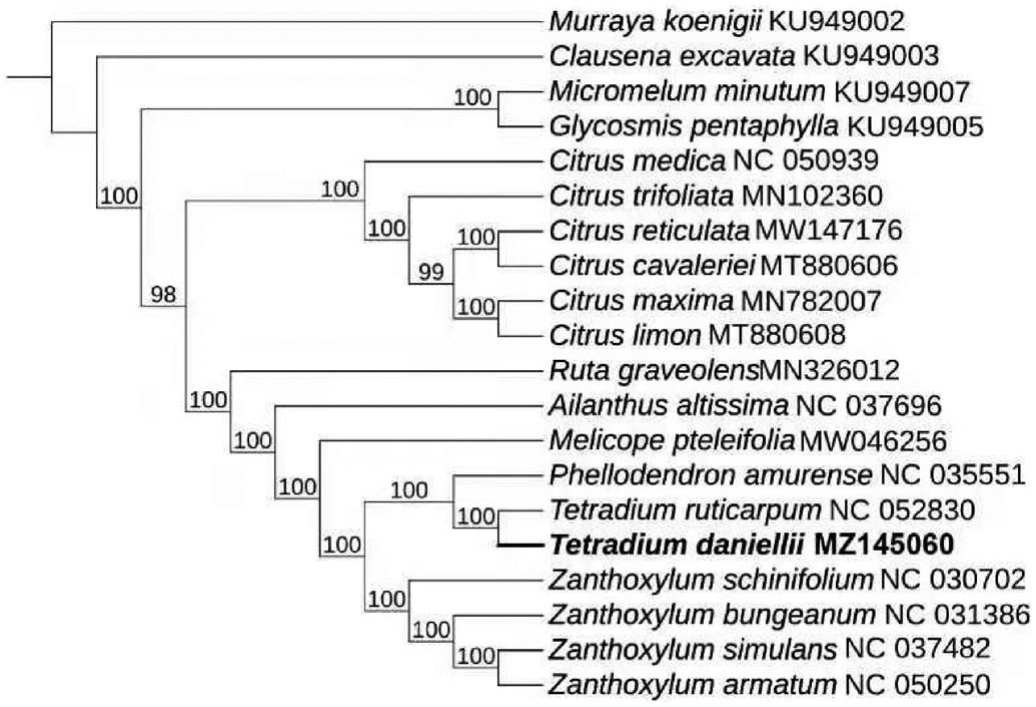
Maximum-likelihood phylogenetic tree for *T. daniellii* based on 20 complete chloroplast genomes.

## Data Availability

The genome sequence data that support the findings of this study are openly available in GenBank of NCBI at (https://www.ncbi.nlm.nih.gov/) under the accession no. MZ145060. The associated BioProject, SRA, and Bio-Sample numbers are PRJNA732282, SRR14663312, and SAMN19316631 respectively.
